# Hemophagocytic Lymphohistiocytosis (HLH) Due to Fulminant Salmonella Sepsis in the Setting of IL12Rβ1 (Interleukin 12 Receptor Beta 1) Deficiency

**DOI:** 10.7759/cureus.41946

**Published:** 2023-07-16

**Authors:** Ali Alabbas, Badriah G Alasmari, Muhammad Saeed, Saeed M Al-Tala, Ayman E Abualama

**Affiliations:** 1 Pediatrics, Najran General Hospital, Najran, SAU; 2 Pediatrics, Armed Forces Hospital South Region - AFHSR, Khamis Mushait, SAU; 3 Pediatric Neurology, Armed Forces Hospital South Region - AFHSR, Khamis Mushait, SAU; 4 Pediatric Genetics, Armed Forces Hospital South Region - AFHSR, Khamis Mushait, SAU; 5 Pediatric Hematology and Oncology, Armed Forces Hospital South Region - AFHSR, Khamis Mushait, SAU

**Keywords:** basille calmette guérin (bcg), salmonella, hemophagocytic lymphohistiocytosis (hlh), mycobacteria, interleukin 12 (il12) il12rβ, mendelian susceptibility to mycobacterial disease ( msmd)

## Abstract

Interleukin 12 receptor beta 1 (IL12Rβ1) deficiency is the most common cause of Mendelian susceptibility to mycobacterial disease (MSMD). MSMD usually predisposes the affected individuals to infections with weakly virulent mycobacteria such as Bacille Calmette-Guérin (BCG), environmental mycobacteria, non-typhoidal Salmonella, and certain other intracellular pathogens. MSMD usually presents with disseminated BCG infection after exposure to the BCG vaccine. Infections with non-typhoidal Salmonella are considered the second most common manifestation of MSMD; however, severe presentation with such organisms is unusual. In this report, we describe a case of a previously healthy infant who was found to have IL12Rβ1 deficiency after she presented with hemophagocytic lymphohistiocytosis (HLH) secondary to severe Salmonella enterica sepsis.

This case report highlights the importance of considering the diagnosis of MSMD in any patient presenting with severe non-typhoidal Salmonella infections even in the absence of any exposure to low-virulent mycobacteria.

## Introduction

Interleukin 12 (IL-12) is an immunoregulatory cytokine secreted by antigen-presenting cells (mainly activated macrophages and dendritic cells). IL-12 has a major role in cell-mediated immunity against intracellular pathogens through the secretion of interferon-gamma (IFN-γ) [1]. Defects in IFN-γ cell-mediated immunity are the causative mechanism of MSMD, which selectively predisposes the affected individuals to infections with weakly virulent intracellular pathogens such as the live attenuated vaccine form of Mycobacterium bovis found in Bacille Calmette-Guérin (BCG) vaccine, environmental mycobacteria, non-typhoidal Salmonella, fungi, Leishmania and also certain viruses [1]. IL-12 has two receptors: interleukin 12 receptor beta 1 (IL12Rβ1) and IL12Rβ2 [2]. IL12Rβ1 deficiency is the most common genetic etiology of MSMD [2]. MSMD usually presents with disseminated BCG infection, which usually develops before one year of age due to the administering of the BCG vaccine at birth [3].

This case report discusses an unusual presentation of MSMD as secondary hemophagocytic lymphohistiocytosis (HLH). HLH is a rare life-threatening hyperinflammatory syndrome caused by aberrantly activated macrophages and cytotoxic T-cells, which can rapidly progress to terminal multi-organ failure [4]. It has two forms, a primary form, which is usually familial with genetic inheritance, and a secondary form, which is usually attributed to various etiologies including infections, hematological malignancies, and others [5].

## Case presentation

The patient was a 17-month-old Saudi girl born at full term to non-consanguineous parents from the southern region of Saudi Arabia. The pregnancy and the delivery had been uneventful. The mother had two healthy children. The previous medical history was unremarkable apart from missing the BCG vaccine. The patient remained healthy until the age of seven months when she developed a high-grade fever associated with decreased activity and poor oral intake for seven days prior to the presentation to the emergency department room of the Armed Forces Hospital of Southern Region (AFHSR), Saudi Arabia. She had been evaluated initially at a private clinic and given oral antibiotics for several days but had failed to show any improvement.

On arrival at the emergency department, she was febrile (39 °C), with oxygen saturation of 88-89% on room air with tachypnea, intercostal and subcostal retractions, with delayed capillary refill, hypotension, and hepatosplenomegaly (liver 5 cm below costal margin and spleen 3 cm below costal margin). There was no associated lymphadenopathy, and the rest of the clinical examination was unremarkable. In the emergency room, she received three boluses of normal saline to correct her hypotension, and her oxygen saturation was maintained above 94% initially with 2 liters of oxygen through a nasal cannula. She also received her first dose of broad-spectrum intravenous antibiotic (ceftriaxone) after obtaining samples for the initial blood routine work and microbiological cultures. The patient was stabilized, and she was shifted to the pediatric intensive care unit (PICU) with a clinical diagnosis of septic shock. Her initial blood investigations revealed a picture of multi-organ impairment in the form of cytopenia, elevated cardiac markers, elevated liver transaminases, elevated renal profile, and coagulopathy (Table 1). Polymerase chain reaction (PCR) for respiratory viral panels including severe acute respiratory syndrome coronavirus 2 (SARS‑CoV‑2) were negative. The initial blood gas was unremarkable. Her initial chest X-ray showed mild bilateral perihilar infiltrates, and the initial echocardiogram showed mild pericardial effusion with normal systolic function. Blood tests for malaria, Leishmania, and serology for Epstein-Barr virus and cytomegalovirus were negative. Due to the high possibility of malignancy, the hematology team was on board from the beginning.

**Table 1:** Lab results of the patient Initial labs showed multiorgan impairment in comparison to the lab results that show significant recovery from HLH. Also, microbiological laboratory results show positive blood culture for Salmonella enterica (Gram-negative rods) and peritoneal fluids positive for yeast cells - Candida spp. HLH: hemophagocytic lymphohistiocytosis; TWBC: total white blood cell count; ANC: absolute neutrophil count; ALT: alanine transaminase; AST: aspartate aminotransferase; GGT: gamma-glutamyl transferase; ALP: alkaline phosphatase; INR: international normalized ratio; APTT: activated partial thromboplastin time; CSF: cerebrospinal fluid

Lab test	Initial lab result	Lab results after recovery	Reference range
Hemoglobin	9.1 g/dL	15.9 g/dL	10–14 g/dl
TWBC	12.7 × 10^9^/L	-	6-17 × 10^9^/L
Platelets	34 × 10^9^/L	464 × 10^9^/L	150–450 × 10^9^/L
ANC	-	2.8 × 10^9^/L	1.8–8 × 10^9^/L
Sodium	156 mmol/L	133 mmol/L	131–145 mmol/L
Potassium	2.5 mmol/L	5.1 mmol/L	3.6–5.5 mmol/L
Urea	49.1 mmol/L	7.09 mmol/L	1.79–9.64 mmol/L
Creatinine	188.5 units/L	32.7 units/L	27–53 units/L
Total protein	51 g/L	-	43–69 g/L
Total bilirubin	119.4 µmol/L	-	<34.2
Direct bilirubin	75.5 µmol/L	-	1.7–8.6 µmol/L
Albumin	16 g/L	-	35–50 g/L
ALT	519 U/L	23 U/L	10–32 U/L
AST	1,574 U/L	36 U/L	18–63 U/L
GGT	160 U/L	10 U/L	5–40 U/L
ALP	1061 U/L	273 U/L	60–321 U/L
Troponin	596 pg/ml	17.1 pg/ml	8.4–18.3 pg/ml
Prothrombin	27.3 seconds	12.6 seconds	12–15 seconds
INR	2.31	1.02	<1.4
APTT	38.6 seconds	29.2 seconds	25–42 seconds
Fibrinogen	109 mg/dL	364 mg/dL	150–400 mg/dL
Ferritin	13,731 µg/L	202 µg/L	13.3–191.9 µg/L
Triglycerides	6.3 mmol/L	1.16 mmol/L	0.45–1.71 mmol/L
Malaria thick film	Negative	-	-
Malaria thin film	Negative	-	-
Hepatitis A	Non-reactive	-	-
Hepatitis B	Non-reactive	-	-
Hepatitis C	Non-reactive	-	-
Urine analysis	Normal	-	-
Urine culture	No growth	-	-
Blood culture	Salmonella enterica (Gram-negative rods)	-	-
CSF WBC	1 HPF	-	0-5 HPF
CSF RBC	2 HPF	-	0-20 HPF
CSF Gram stain	No organism seen	-	-
CSF culture	No growth	-	-
Peritoneal fluid WBC	64 × 10^9^/L	-	0-20 × 10^9^/L
Peritoneal fluid RBC	85 × 10^12^/L	-	0-20 × 10^12^/L
Peritoneal fluid Gram stain	No organism seen	-	-
Peritoneal fluid culture	Yeast cell - Candida spp.	-	-
Brucella titer	1/80	-	1/80

The patient fulfilled six out of eight HLH 2004 diagnostic criteria [4]. She had a fever, splenomegaly, cytopenia, high ferritin, high triglycerides, and low fibrinogen. The hematology team decided to investigate for lymphoma with a pan CT scan, which came back normal, and the patient subsequently underwent a bone marrow biopsy. She was supported with packed red blood cells and platelet transfusions before undergoing the bone marrow biopsy (Table 2). The patient's first blood culture (within 24 hours of admission) revealed Gram-negative rods, which were later identified as Salmonella enterica. Her urine and cerebrospinal fluid cultures showed no growth. Blood samples for hemoglobin electrophoresis, flow cytometry, and whole exome sequencing (WES) study were taken as there was a concern for immune deficiency and given the high prevalence of sickle cell disease in the southern region of Saudi Arabia (Tables 1, 2).

**Table 2:** HLH laboratory results HLH: hemophagocytic lymphohistiocytosis; WES: whole exome sequencing

Lab test	Lab value	Reference range
Hemoglobin	7 g/dL	10–14 g/dl
Platelets	7 × 10^9^/L	150–450 × 10^9^/L
Ferritin	13,731 µg/L	13.3–191.9
Triglycerides	6.1 mmol/L	0.45–1.71
Fibrinogen	109 mg/dL	150–400
Bone marrow aspiration	Hemophagocytosis with features of sepsis, no evidence of malignancy	-
NK activity	Not done	-
Genetic test	IL12Rβ1 (NM-005535.3)	-
WES	IL12Rβ1 C1272del P. (Glu425 Argfs *30) chr19; 18179253 pathogenic	-
Peripheral blood smear	Hypochromic microcytic anemia suggestive of iron deficiency anemia; toxic granulation and vacuolation of neutrophils suggestive of infection and severe thrombocytopenia with normal-sized platelets	-

Initially, the patient was given two doses of intravenous immunoglobulins and continued on intravenous ceftriaxone with the addition of vancomycin as well as multiple packed RBC transfusions and platelet transfusions. Her condition deteriorated during the first few days of admission and she had a persistent fever; hence, ceftriaxone was upgraded to meropenem. Also, she had aggravated respiratory distress with increased oxygen requirement with features of acute respiratory distress syndrome in the repeated chest X-ray and required mechanical ventilation for four days, which was then weaned off and replaced with high-flow nasal cannula oxygen. In addition, her renal functions deteriorated, and hence she was started on peritoneal dialysis, which failed initially and she, therefore, required hemodialysis. After a few days, hemodialysis was stopped and peritoneal dialysis was resumed successfully. Her associated hypertension was controlled with hydralazine and amlodipine.

The patient's peripheral blood smear showed hypochromic microcytic anemia suggestive of iron deficiency anemia, toxic granulation, and vacuolation of neutrophils suggestive of infection and severe thrombocytopenia with normal-sized platelets. The bone marrow biopsy revealed hemophagocytosis with features of sepsis and no evidence of malignancy. Therefore, HLH was confirmed and the patient was placed on HLH 2004 treatment protocol, initially with intravenous dexamethasone (10 mg/m2/day) on the third day of admission with the addition of etoposide on the sixth day of admission (with 50% reduction of the initial dose as adjustment for the renal impairment) (Tables 1, 2).

The patient's condition started to improve gradually. The fever and coagulopathy resolved, her respiratory condition improved, and she was weaned off from oxygen requirement gradually to room air over a few days of admission. Also, her renal function and hypertension showed gradual improvement and, eventually, the dialysis and antihypertensive drugs were stopped. Her deranged liver function impairment normalized and her hemoglobin level and platelet counts stabilized. The repeated blood and urine cultures showed no growth. The intravenous antibiotics were continued for a total of two weeks. Later, hemoglobin electrophoresis and flow cytometry came back normal. The WES study revealed a homozygous pathogenic variant of IL12Rβ1, which was consistent with the diagnosis of rare autosomal recessive MSMD.

The patient was discharged home on the 17th day of admission in good condition with advice to follow up with hematology to complete her HLH protocol and nephrology clinics. Three days later, she was readmitted to the pediatric ward due to a yellowish discharge from the peritoneal dialysis catheter with no other associated features of active infection. Peritoneal fluid analysis revealed yeast. Therefore, the patient was treated for fungal peritonitis. However, blood and urine cultures showed no growth. The peritoneal dialysis catheter was removed and she was treated with amphotericin B for three weeks while on HLH protocol. The fungal infection resolved completely and she was sent home again in good condition.

Thereafter, she was monitored in the hematology clinic and completed three weeks of dexamethasone (tapered in the third week) and eight weeks of etoposide therapy. Afterward, she was evaluated at week nine since the start of her treatment and showed non-active disease, and hence the treatment was stopped (Table 1). Currently, she is two and half years old and in a good healthy condition; she has been out of her crisis for more than a year and continues to regularly follow up with pediatric hematology and oncology.

## Discussion

IL12Rβ1 deficiency is the most common cause of MSMD [6-7]. It usually presents with disseminated Mycobacterium bovis infection following exposure to BCG vaccine [3]. Our patient did not receive the BCG vaccine due to the recent change in the Saudi Ministry of Health's national vaccination program, which recommended delaying the BCG vaccine tile the age of six months for infants. This new recommendation was based on the high prevalence of primary immune deficiency disorders in Saudi Arabia, which is related to the high frequency of consanguinity [8].

Infection with Salmonella is considered the second most common manifestation of MSMD [3], but the severe presentation of non-typhoidal Salmonella is unusual. Our patient presented with a severe manifestation of non-typhoidal Salmonella (Salmonella enterica) with multi-organ impairment and secondary HLH. We conducted a thorough search of the existing literature, and, to the best of our knowledge, there were no reported cases of HLH secondary to infections with non-typhoidal Salmonella. On the contrary, HLH secondary to typhoidal Salmonella infection has been reported in a few cases. One case report described a previously healthy four-year-old girl with HLH secondary to Salmonella typhi [9] and another case report discussed a rare presentation of typhoid fever with meningitis and secondary HLH in a previously healthy five-year-old child [10]. Both patients in the above-mentioned case reports had no evidence of immune deficiencies [9-10]. The severe presentation in our patient seems to be due to the underlying IL12Rβ1 deficiency, which selectively predisposes the affected individual to infections with non-typhoidal Salmonella among other intracellular pathogens [3].

## Conclusions

This case report emphasizes the importance of screening for MSMD in any patient with a severe presentation of non-typhoidal Salmonella infection. Furthermore, this report highlights the importance of delaying the BCG vaccine until screening for MSMD has been done, especially in communities with a high frequency of consanguinity and a high prevalence of primary immune deficiency disorders.
